# Biocatalytic 3D binary crystals formed through the self-assembly of enzyme-embedded ferritin

**DOI:** 10.1039/d5nr03463a

**Published:** 2025-11-04

**Authors:** Yu Zhou, Lotta Rosenlöf, Boxuan Shen, Mauri A. Kostiainen

**Affiliations:** a Biohybrid Materials, Department of Bioproducts and Biosystems, Aalto University 00076 Aalto Finland mauri.kostiainen@aalto.fi; b School of Chemistry, Xi'an Key Laboratory of Sustainable Energy Material Chemistry, Xi'an Jiao Tong University 710049 Xi'an People's Republic of China; c Department of Medical Biochemistry and Biophysics, Karolinska Institutet 17177 Stockholm Sweden

## Abstract

Protein crystals are traditionally used to aid structural analysis but have recently gained attention as functional materials due to their intrinsic order, defined porosity, and high chemical programmability. While enzymes have been incorporated into protein crystals, most existing systems rely on post-crystallization loading or nonspecific adsorption, offering limited control over the spatial distribution of catalytic components. Furthermore, there are few examples of catalytically active crystals formed through the ordered assembly of protein nanocages. These modular and uniform building blocks provide precise size, surface chemistry, and cargo loading, making them ideal candidates for constructing functional crystalline materials. In this study, we report a binary protein crystal formed by electrostatic co-crystallization of oppositely charged ferritin nanocages, with one component encapsulating the peroxidase-mimicking enzyme enhanced ascorbate peroxidase 2 (APEX2). The resulting material exhibits long-range order, retains enzymatic activity, and can be reused in multiple catalytic cycles. This platform provides a framework for building multienzyme crystalline assemblies and studying spatially programmed biocatalysis.

## Abstract 2

Enzyme-embedded protein cage crystals are formed via electrostatic co-crystallization, enabling reusable biocatalytic assemblies with spatial control.

## Graphical abstract

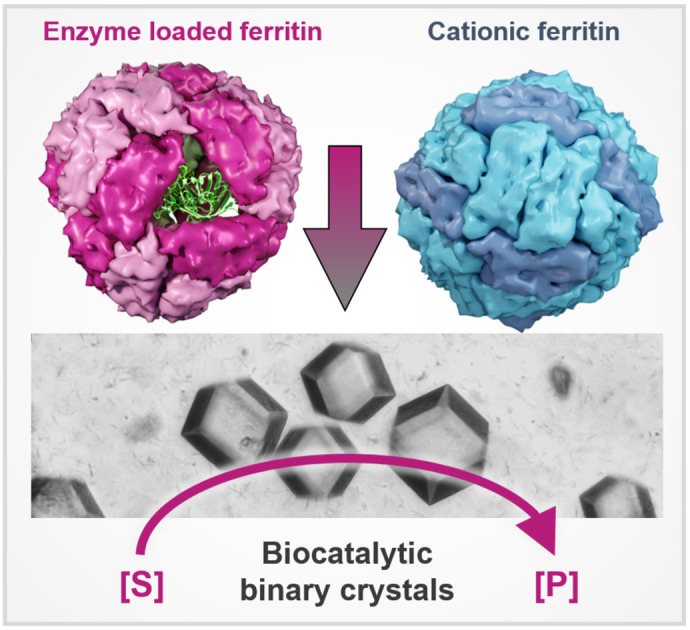

## Introduction

Advances in protein engineering have enabled the design of functional protein-based materials with applications in catalysis, sensing, and nanofabrication.^1–4^ Among these, protein crystals represent a distinct class of solid-state biomaterials, characterized by molecular-level order, high porosity, and tunable surface chemistry.^5,6^ Originally developed for structural biology,^7^ they are now gaining attention as functional scaffolds that translate molecular properties into macroscopic performance.

The highly defined structure, porosity and tunable surface chemistry make protein crystals well-suited for catalysis. Specifically, their crystalline architecture offers key advantages for catalytic applications: high porosity facilitates substrate diffusion to catalytic sites, structural robustness enhances operational stability, and compatibility with chemical or genetic modification that allows customization of catalytic functions.^8,9^ Their crystalline architecture facilitates also enzyme immobilization, enhances reusability, and preserves catalytic function under harsh conditions. These attributes indicate that protein crystals function beyond passive supports and can serve as actively designable frameworks for enzyme-based catalysis.^10–12^

To date, most efforts have focused on porous protein crystals, which stabilize embedded enzymes and allow substrate diffusion through internal channels.^9,10,13–18^ However, these systems typically rely on post-crystallization loading or nonspecific immobilization of enzymes, and offer limited control over the spatial distribution, orientation, and loading density of active sites. The lack of programmability hinders the ability to establish a clear correlation between crystalline structure and catalytic performance, thereby limiting the development of accurately organized biocatalytic systems.

Protein cages, such as ferritin and viral capsids like Cowpea Chlorotic Mottle Virus (CCMV), provide a modular platform for nanoscale compartmentalization.^19–23^ Their uniform size, symmetrical structure, and diverse surface chemistry allow precise control over cargo loading and interparticle interactions.^20,24,25^ These properties have enabled for example the construction of ordered metal nanoparticle superlattices and the development of bioactive nanostructures, including enzyme carriers with tunable catalytic environments.^26–35^

Despite these advances, catalytically active crystalline assemblies formed through ordered arrangements of protein cages remain limited,^36^ highlighting a gap in systems that integrate the spatial precision of catalysts with the structural stability of crystalline frameworks. Creating such systems offers a strategy to spatially organize enzymes within defined architectures, enabling cooperative or cascade catalysis with enhanced reusability and performance. In this work, we present a binary protein crystal assembled *via* electrostatic co-crystallization of two engineered ferritin variants. The negatively charged ferritin encapsulates a supercharged peroxidase, APEX2,^37^ while the positively charged variant promotes crystal growth through electrostatic interactions. The resulting crystalline material displays high enzyme loading, long-range order, and retained enzymatic activity across multiple reaction cycles. Notably, this binary co-crystal system faces specific challenges, including scaling up the electrostatic assembly while maintaining crystalline order and ensuring the crystallization process remains stable against minor pH or ionic strength fluctuations. Ultimately, this work establishes a versatile strategy for building functional protein cage crystals and provides a foundation for programmable biocatalytic architectures.

## Results and discussion

**Fig. 1:**
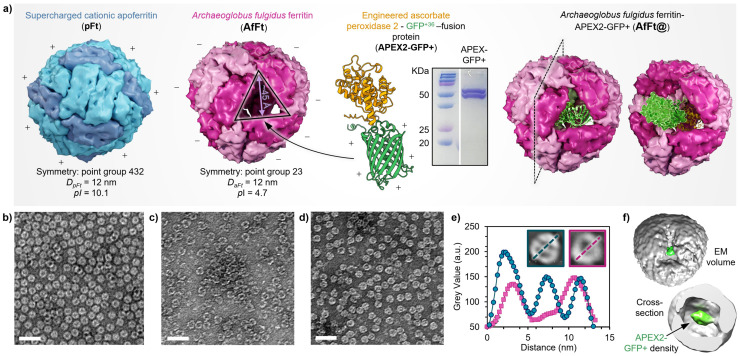
Materials used for the assembly of catalytic protein cage crystals. (a) Constituent building blocks for construction: supercharged cationic apoferritin (pFt), *Archaeoglobus fulgidus* ferritin (AfFt), and Engineered Ascorbate Peroxidase 2-GFP+36 (APEX2-GFP+). (b–d) TEM images of pFt (b), AfFt (c) and AfFt@ (d), scale bars: 20 nm. (e) Mean grey value profile *vs*. distance for AfFt@ and empty AfFt (blue and pink, respectively). Insets show representative 2D class average images, derived from the AfFt@ TEM dataset (d). (f) EM density of AfFt@, showing the encapsulated GFP+ (green) within the internal cavity of AfFt (gray) through the cage pore (top) and cross-section (bottom).

To construct catalytic protein cage crystals, we selected *Archaeoglobus fulgidus* ferritin (AfFt) for its distinct structural and surface charge features that enable efficient protein encapsulation under mild conditions.^38^ Unlike conventional ferritins, AfFt assembles into a tetrahedral 24-mer with large triangular openings (∼4.5 nm) and can reversibly dissociate into dimers at neutral pH and low ionic strength.^39,40^ This allows access to its interior cavity without disrupting the cage. The negatively charged inner surface of AfFt promotes electrostatic encapsulation of cationic cargo.^41^ We encapsulated a fusion protein comprising APEX2 and GFP+36, a highly positively charged green fluorescent protein (GFP+) variant, which facilitates strong interaction with the AfFt interior (Fig. 1a). The enzyme-loaded cages maintain their structure during co-crystallization with a positively charged ferritin variant pFt (Fig. 1b), highlighting the robustness of AfFt as a scaffold for constructing catalytically active, protein cage-based crystals.

AfFt was first recombinantly expressed and purified from *E. coli* (Fig. S1a). The morphology of the empty cage was confirmed by transmission electron microscopy (TEM) under 300 mM NaCl, showing nanocage structures with the expected dimensions (Fig. 1c).^41^ A fusion protein consisting of APEX2 at the *N*-terminus and GFP+36 at the C-terminus, connected by a glycine-serine (GS) linker, was similarly expressed. The recombinant fusion protein was expressed in the apo form and subsequently incubated with excess hemin *in vitro* to achieve cofactor incorporation.^37,42^ The resulting complex was further purified by size-exclusion chromatography (SEC), yielding a product with sufficient heme occupancy (Fig. S1b). To encapsulate the fusion protein, AfFt was mixed with the APEX2-GFP+ at a 1 : 3 molar ratio (one cage to three cargo molecules) under 300 mM NaCl yielding AfFt@. TEM imaging revealed increased internal contrast within the cages, indicating successful cargo loading (Fig. 1d), while calculations based on the UV-vis spectra of AfFt@ samples showed an average of 1.45 cargo molecules per cage. Subsequent single-particle 3D reconstruction confirmed the presence of guest proteins inside the AfFt cavity, 12 461 particles were initially picked from single-particle data and 12 177 were retained after 2D classification. Each 2D class average is labeled with its particle count (Fig. S2), including those in the insets of Fig. 1e: blue-boxed AfFt@ (399 particles) and pink-boxed empty AfFt (26 particles). As for the 3D reconstruction results, the internal density was clearly visible through the characteristic triangular openings, consistent with successful encapsulation (Fig. 1f).

To facilitate crystallization, we engineered a positively charged ferritin variant (pFt) by introducing nine lysine or arginine residues per subunit on the surface of human heavy chain apoferritin, thereby generating a net cationic surface.^43^ When combined with the negatively charged AfFt cages at neutral pH, pFt and AfFt form complexes through electrostatic interactions, promoting the formation of binary protein cage crystals. The strength of the electrostatic interactions can be finely tuned by adjusting the electrolyte concentration in the solution. This charge-driven assembly offers a straightforward and modular approach to construct catalytically active protein cage crystals that exhibit both precise structural organization and integrated functionality.

**Fig. 2:**
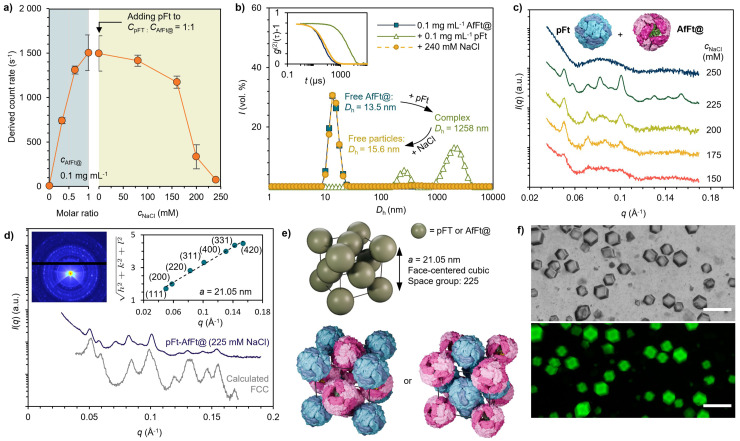
Self-assembly and structural characterization of the catalytic protein cages crystals. (a) Left: light scattering intensity of AfFt@ (0.1 mg mL^−1^) upon the addition of pFt (0.1 mg mL^−1^), indicating complex formation. Right: complexes were disassembled by increasing the ionic strength of the medium through the addition of NaCl. Data is reported as an average of three independent samples with error bars showing the standard deviation. (b) DLS data showing the volume-averaged size distribution of complex formation and disassembly from panel (a). Inset: second order normalized autocorrelation function of all samples. (c) SAXS data measured for the catalytic protein cages crystals at various NaCl concentrations. (d) Two-dimensional (inset) and integrated one-dimensional SAXS data of the catalytic protein cages crystals at 225 mM NaCl, showing the experimental data collected with a hanging-drop setup (deep blue curve), together with the respective calculated scattering pattern of the FCC structure (grey curve; offset vertically for clarity). (e) Proposed FCC unit cell structure of the catalytic protein cage crystals with different possible packing modes. (f) Top: Optical microscopy image of catalytic protein cage crystals formed *via* hanging drop vapor diffusion, with crystal dimensions exceeding 20 μm. Scale bar: 100 μm. Bottom: Corresponding GFP fluorescence image, confirming the presence of GFP-labeled APEX2 enzyme within the crystalline lattice.

Electrostatic interactions play a key role in assembling protein cages into higher order structures, and our previous studies have demonstrated that precise modulation of these interactions through electrolyte concentration is an effective strategy for controlling nanoparticle organization.^44–48^ To explore the salt-dependent assembly behavior of catalytic protein cage crystals, we employed dynamic light scattering (DLS) to monitor changes in scattering intensity (derived count rate) and hydrodynamic diameter (*D*_h_). In this assay, positively charged pFt (0.1 mg mL^−1^) was added to a Tris buffer (20 mM Tris, pH 7.4) containing negatively charged AfFt@ (0.1 mg mL^−1^). Upon pFt addition, a marked increase in count rate was observed, indicating the rapid formation of large supramolecular assemblies. The contribution of electrostatic attraction was verified by titrating NaCl into the solution (Fig. 2a). Increasing the ionic strength progressively screened electrostatic interactions by shortening the Debye length, leading to a gradual reduction in complex size. At approximately 240 mM NaCl, both the scattering intensity and *D*_h_ returned to values comparable to free AfFt@ and pFt, indicating complete disassembly of the supramolecular complexes and recovery of the individual cages (Fig. 2b and Fig. S2).

Next, small-angle X-ray scattering (SAXS) was employed to study the structural organization of protein cage assemblies formed under different NaCl concentrations. At low ionic strength (*e.g.*, 150 mM NaCl), the scattering profile was featureless, indicating amorphous aggregation. At high salt concentration (250 mM NaCl), the SAXS pattern showed only the form factor of free particles, consistent with complete disassembly as confirmed by DLS. In contrast, at intermediate salt concentrations (175, 200, and 225 mM), distinct Bragg reflections were observed in the SAXS profiles, indicating ordered assembly. Notably, at 225 mM NaCl, the two-dimensional SAXS images exhibited multiple concentric Debye rings, indicative of polycrystalline structures with isotropic orientation. Azimuthally integrated scattering curves showed multiple sharp diffraction peaks, consistent with long-range crystalline order (Fig. 2c). The prominent diffraction peaks observed at 225 mM appeared at *q* of 0.501, 0.582, 0.826, 1.012, 1.304, 1.426, and 1.534 nm^−1^. These were indexed to the (111), (200), (220), (311), (400), (331), and (420) planes, respectively, corresponding to *q*^*n*^/q* ratios of √3, √4, √8, √11, √16, √19, and √20. A linear fit of *q*(*hkl*) *versus* √(*h*^2^ + *k*^2^ + *l*^2^) yielded a lattice constant of *a* = 21.05 nm. Coupled with comparison to simulated scattering curves of a finite face-centered cubic (FCC) structure, these results confirm that AfFt@ and pFt cages adopt an FCC Bravais lattice with space group *Fm*3̄*m* (No. 225) (Fig. 2d). Given that protein cage particles bearing like charges would be expected to repel each other, we hypothesize that the AfFt@–pFt crystals adopt an AB_3_-type unit cell arrangement to accommodate favorable packing (Fig. 2e). Notably, highly ordered face-centered cubic (FCC) crystals with a characteristic rhombic dodecahedral habit, reaching up to 50 μm in length, were rapidly formed within 4 hours using a hanging drop method under 225 mM NaCl. Here, the rhombic dodecahedral habit in FCC crystals is a result of the crystal's tendency to minimize its surface energy by growing in the direction of the {100} and {111} facets leaving a habit enclosed by the {110} faces.^36,49^ The corresponding GFP channel fluorescence image confirms the successful incorporation of the APEX2-GFP+ fusion protein within the crystalline lattice, demonstrating the spatial organization of functional protein cargos throughout the crystal structure (Fig. 2f).

**Fig. 3:**
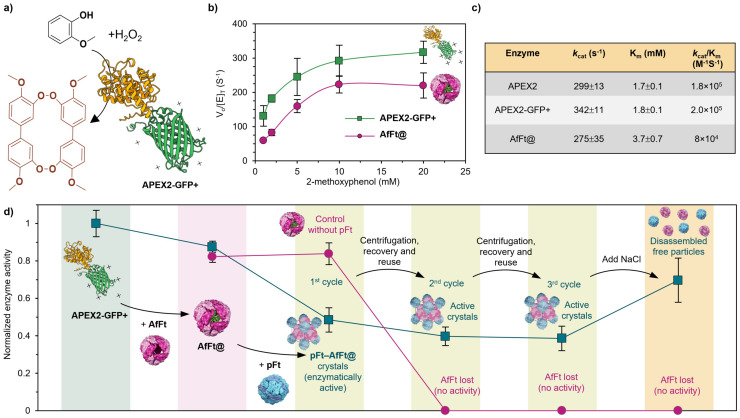
Catalytic properties of the AfFt@–pFt crystals. (a) Schematic illustration of the APEX2-GFP+ catalyzed 2-methoxyphenol oxidation reaction. (b) Michaelis–Menten kinetics of APEX2-GFP+ and AfFt@, from which kinetic parameters were derived. (c) Extracted kinetic parameters using 2-methoxyphenol as the substrate; *K*_m_ values reflect affinity toward 2-methoxyphenol. (d) Comparative analysis of catalytic activity across different assembly configurations. Enzymatic activity is quantitatively normalized to free APEX2-GFP+, which is set as 1.

Building on the insights from structural analysis, we proceeded to evaluate the catalytic performance and reusability of the AfFt@–pFt crystalline assemblies. We began by examining the activity of the fusion enzyme APEX2-GFP+ alone using a 2-methoxyphenol oxidation assay.^42^ In the presence of hydrogen peroxide, APEX2 catalyzes the oxidation of 2-methoxyphenol to a chromogenic product with a characteristic absorbance at 470 nm (Fig. 3a and Fig. S4). Under constant H_2_O_2_ concentration (1 mM), 2-methoxyphenol was titrated from 0.25 to 20 mM, and kinetic parameters (*k*_cat_ and *K*_m_) were derived *via* Michaelis–Menten fitting (Fig. 3b). The results indicated that APEX2-GFP+ retained catalytic efficiency comparable to that of native APEX2, suggesting that fusion to GFP+ did not significantly affect enzymatic activity.^37^ Following encapsulation within AfFt cages *via* electrostatic interaction, the resulting AfFt@ cage exhibited slightly reduced *k*_cat_, with 80.4% of the activity of the free fusion protein. Importantly, the encapsulated enzyme remained catalytically competent, indicating successful loading without significant loss of function (Fig. 2c).

Next, we co-assembled AfFt@ with pFt under crystallization conditions to yield catalytic protein cage crystals. In the initial catalytic assay, crystals containing equivalent total enzyme amount exhibited a relative activity of 0.41 ± 0.06 compared to free APEX2-GFP+ (Fig. 3d). This decrease is attributed to heterogeneous catalysis, where substrate diffusion through the solid lattice is more restricted than in solution. Nonetheless, the solid-state format offers key advantages, including facile separation and reuse of the catalytic material.^50,51^ To assess reusability, the crystal-containing reaction mixtures were subjected to centrifugation to remove supernatant, followed by the addition of fresh substrate-containing solution. As shown, the catalytic activity remained stable over multiple consecutive cycles, demonstrating robust performance and recyclability. Notably, upon the addition of excess NaCl, the crystals disassembled, and enzymatic activity partially recovered to 0.623 ± 0.118, consistent with the release of individual AfFt@ cages back into solution. In contrast, AfFt@ alone, which lacks the crystalline scaffold, could not be recycled: once centrifuged and resuspended in fresh substrate, no catalytic activity was detected. These findings underscore the advantage of the AfFt@–pFt crystals as a heterogeneous catalytic platform, combining structural order with effective enzyme stabilization and reusability.

## Conclusion

In summary, we present a modular protein cage co-crystallization strategy that enables the spatial organization of enzymatically active cargo within an ordered crystalline lattice. By electrostatically assembling negatively charged AfFt cages loaded with APEX2-GFP+ fusion proteins and positively charged pFt units, we generated face-centered cubic (FCC) protein cage crystals that retain catalytic activity and exhibit excellent recyclability as heterogeneous catalysts. Notably, pFt not only serves as a structural counterion but also allows encapsulation of functional cargos such as metal oxide nanozymes, offering a route to build native enzyme–nanozyme cascade systems within a unified crystalline architecture. In future, such a design allows precise spatial positioning of distinct catalytic components, enabling tunable interparticle distances and controlled reaction environments. The spatial control provides a promising platform for probing multistep biocatalysis and artificial metabolic architectures. Practical implications may include for example biosensing where the ordered crystalline lattice can enhance signal transduction by guiding the diffusion of reactive species, thus increasing the sensitivity of conventional enzyme-based sensors. In bioremediation, the crystalline structure may provide protection for enzymes in harsh solution conditions and enable biocatalyst recycling. In synthetic biology, such ordered structures can support cascade catalysis by enabling control over enzyme spacing. Together, these attributes position biocatalytic enzyme crystals to bridge laboratory scale designs and practical applications.

## Conflicts of interest

There are no conflicts of interest to declare.

## Supplementary Material

NR-017-D5NR03463A-s001

## Data Availability

The data supporting the findings of this study are available within the article and its supplementary information (SI). Supplementary information is available. See DOI: https://doi.org/10.1039/d5nr03463a.
