# Supplementary material for: Biocatalytic 3D binary crystals formed through the self-assembly of enzyme-embedded ferritin
Source: Nanoscale. 2025 Nov 4;17(45):26411–6. doi: 10.1039/d5nr03463a (PMC12590944; doi:10.1039/d5nr03463a)
Supplement: NR-017-D5NR03463A-s001 [file NR-017-D5NR03463A-s001.pdf]

Supplementary Information (SI) for Nanoscale.
This journal is © The Royal Society of Chemistry 2025

Supplementary information

Biocatalytic 3D Binary Crystals Formed Through the Self-Assembly of Enzyme-Embedded Ferritin

Yu Zhou[1,2], Lotta Rosenlöf[1], Boxuan Shen[3] and Mauri A. Kostiainen[1,]*

[1] Biohybrid Materials, Department of Bioproducts and Biosystems, Aalto University, 00076 Aalto, Finland. E-mail: mauri.kostiainen@aalto.fi

[2] School of Chemistry, Xi'an Key Laboratory of Sustainable Energy Material Chemistry, Xi'an Jiao Tong University, 710049 Xi'an, People's Republic of China

[3] Department of Medical Biochemistry and Biophysics, Karolinska Institutet, 17177 Stockholm, Sweden

MATERIALS AND METHODS

Materials. Glass microscope slides and cover slips were obtained from VWR. Hemin chloride, 30% hydrogen peroxide and the other chemicals were sourced from Sigma Aldrich, unless otherwise specified. MilliQ water with a resistivity greater than 18.2 MΩ·cm was used for all experiments.

Protein Expression and Purification. The protein backbone sequences, namely pFt, AfFt, and APEX2-GFP+, were initially optimized using OptimumGene™ and subsequently synthesized and cloned into the pET-25b(+) vector by GenScript. *E. coli* T7 Express cells (New England Biolabs) were used as the expression host, and protein expression and purification followed a previously established protocol. To maximize hemin incorporation, purified APEX2-GFP+ was reconstituted with hemin chloride by mixing Ni-NTA and ion exchange (HiTrap Heparin HP) purified protein with an equimolar amount of hemin chloride dissolved in 10 mM NaOH, followed by incubation at room temperature for 1 hour. The reconstituted protein was further purified by size-exclusion chromatography (Superdex 200 10/300 GL, GE Healthcare Life Sciences) at a flow rate of 0.5 mL/min using PBS (pH 7.4) as the mobile phase. Eluted fractions were monitored at 260, 280, and 408 nm, collected, and analyzed by SDS-PAGE. When necessary, protein samples were concentrated using Amicon Ultra-15 centrifugal filter units with a 10 kDa molecular weight cutoff (Merck Millipore). Protein purity was evaluated by SDS-PAGE (Fig.

S1), and concentrations were determined using a NanoDrop LITE spectrophotometer (Thermo Scientific) based on absorbance at 405 nm, using an extinction coefficient of 128,000 M^{-1} cm^{-1}. Final protein samples were flash-frozen in liquid nitrogen and stored in aliquots at −80 °C.

Dynamic light scattering (DLS). The measurements were performed using a Zetasizer Nano ZS instrument (Malvern Instruments) to determine the hydrodynamic diameter (D_h) of the assemblies. The instrument was equipped with a 4 mW He–Ne laser (λ = 633 nm) and an avalanche photodiode detector positioned at a backscattering angle of 173°. All measurements were conducted at 25 °C using PMMA cuvettes. Data analysis, including scattering intensity (count rate) and particle size distribution, was carried out using Zetasizer software (Malvern Instruments).

In the experiments, AfFt@ (0.1 mg mL^{-1}) was first added to a Tris buffer solution (20 mM Tris, pH 7.4), followed by the sequential addition of the pFt solution.

Small angle X-ray scattering (SAXS) experiments. The measurements were carried out using a Xenocs Xeuss 3.0 C system, featuring a GeniX 3D Cu microfocus X-ray source (λ = 1.542 Å) and an EIGER2 R 1M hybrid pixel detector. The sample-to-detector distance was set to 1.1 m. Two-dimensional scattering patterns were azimuthally averaged to obtain one-dimensional SAXS profiles. The scattering vector q was calculated using the equation $q = 4\pi \sin\theta / \lambda$, where 2θ is the scattering angle.

For sample preparation, 6 μL of NaCl-containing buffer (20 mM Tris-HCl, pH 7.4) was added to 1 μL of AfFt@ (final concentration: 2 mg mL^{-1}) to modulate the ionic strength. Subsequently, 1 μL of pFt solution (final concentration: 2 mg mL^{-1}) was introduced under continuous stirring.

Electron microscopy imaging. Transmission electron microscopy (TEM) was employed to visualize individual protein cages using a Tecnai 12 Bio-Twin microscope (FEI) operated at an acceleration voltage of 120 kV. Samples were deposited onto Formvar/carbon-coated copper grids, and excess solution was blotted with filter paper. The grids were then rinsed with Milli-Q water, followed by staining with 2% uranyl acetate for 30 seconds.

Single-particle reconstruction. Single-particle analysis was performed using CryoSPARC to reconstruct the 3D structure of the protein cages. Contrast transfer function (CTF) estimation were carried out using Patch CTF Estimation. Micrographs with poor CTF fits or visible artifacts were discarded. Particles were automatically picked using the blob picker or template-based picking (depending on data quality), followed by several rounds of 2D classification to remove false positives and damaged particles.

High-quality particle subsets were then used for ab initio model generation and heterogeneous refinement. The best class was selected for non-uniform refinement to obtain the final 3D reconstruction. All steps were conducted using default parameters unless otherwise specified. The resolution of the final map was estimated based on the gold-standard Fourier shell correlation (FSC = 0.143) criterion.

Optical microscopy. Samples for optical microscopy were prepared using the hanging drop method. An 18 × 18 mm cover slip (VWR) was used to suspend a droplet above a reservoir containing 300 μL of 20 mM Tris buffer (pH 7.5). The droplet composition matched that used in the SAXS experiments. To minimize solvent evaporation, the cover slip was affixed to the reservoir cap with high vacuum grease, positioning the droplet toward the sealed interior. The assembled samples were incubated at room temperature prior to imaging.

Microscopy was performed using a Zeiss Axio Vert A1 inverted microscope. Imaging was carried out directly on the glass slides employed during crystallization, without further sample processing.

Enzyme activity test. 2-methoxyphenol assays were performed using an Agilent Cary 60 UV-Vis spectrophotometer. Equal concentrations of purified, heme-bound holoenzyme were used across all conditions, including free enzyme, AfFt-encapsulated enzyme (AfFt@), and crystalline forms. Enzyme concentrations were calculated based on the heme-bound protein content, determined by absorbance at 405 nm using an extinction coefficient of 128,000 M^{-1} cm^{-1}.

2-methoxyphenol (Sigma) was prepared in PBS (pH 7.4) at room temperature to final concentrations ranging from 0.25 to 20 mM. Solutions were incubated at 37 °C for at least 5 minutes and vortexed thoroughly to ensure complete dissolution. Hydrogen peroxide (H_2O_2, Sigma) was freshly diluted from stock to a working concentration of 1 mM.

Reactions were assembled in disposable plastic cuvettes by combining 1 mL of 2-methoxyphenol solution with 1 μL of H_2O_2. Reactions were initiated by the addition of 1–2 μL of enzyme stock solution, followed by immediate manual mixing. The formation of tetraguaiacol was monitored by measuring absorbance at 470 nm. Reaction rates were calculated using an extinction coefficient of $\varepsilon_{470} = 22$ mM^{-1} cm^{-1}.

Kinetic parameters (k_{cat} and K_m) were determined by fitting initial velocity data to the Michaelis–Menten equation using nonlinear regression in Origin. Standard errors were derived from the variance–covariance matrix and the mean residual variance, as calculated by the software.

Enzyme Reusability Assay. As described above, the concentration of APEX2 in both the free fusion enzyme and AfFt@ samples were determined based on the absorbance at 405 nm, corresponding to the heme cofactor. For the pFt–AfFt@ crystal samples, the enzyme concentration was estimated indirectly. After assembly and sedimentation, the supernatant was collected and analyzed via the 2-methoxyphenol assay to quantify the unassembled AfFt@ fraction. The enzyme content within the crystals was calculated by subtracting the amount in the supernatant from the total amount of enzyme initially added to the assembly mixture.

In the reusability assay, after each enzymatic activity measurement, the pFt–AfFt@ crystal samples were centrifuged at 2000 rpm for 1 minute to pellet the crystals. The supernatant was completely removed, and fresh assay solution was added for the next cycle of activity measurement. As a control, samples were prepared under identical conditions except that the pFt component was omitted during

assembly. This prevented crystal formation, allowing the enzyme to remain in solution, and served as a non-immobilized reference.

Protein Sequences. The pFt sequence used in this study was derived from FtnQC12, with nine mutated residues highlighted in red.

MTTASTSQVRQNYHQDSEKAINRQIRLELYASYVYLSMSYYFDRDDVALKNFAKYFLHQSHE
EREHAEKLMKLQNQRGGRIFLQDIQKPDKDDWESGLRAMEKALKLEKKVNQSLLELHKLAT
KKNDPHLCDFIETHYLNEQVKAIKELGDHVTNLRKMGAPRSGLAEYLFDKHTLGDSDNES

AfFt sequence:

MASISEKMVEALNRQINAEIYSAYLYLSMASYFDSIGLKGFSNWMRVQWQEELMHAMKMF
DFVSERGGRVKLYAVEEPPSEWDSPLAAFEHVYEHEVNVTKRIHELVEMAMQEKDFATYNF
LQWYVAEQVEEEASALDIVEKLRLIGEDKRALLFLDKELSLRQFTPPAEEEK

APEX2-GFP+ sequence:

MHHHHHHMGKSYPTVSPDYQDAIEKAKRKLRGFIAEKKCAPLILRLAFHSAGTFDSKTKTGG
PFGTIKHQAELAHGANNGLDIAVRLLEPIKEQFPIVSYADFYQLAGVVAVEITGGPKVPFHPG
REDKPEPPPEGRLPDATKGSDHLRDVFGKAMGLSDQDIVALSGGHTIGAAHKERSGFEGPWT
SNPLIFDNSYFTELLTGEKDGLLQLPSDKALLTDSVFRPLVEKYAADEDVFFADYAEAHLKLS
ELGFAEAGGGSGGGSMASKGERLFRGKVPILVELKGDVNGHKFSVRGKGKGDATRGKLTLK
FICTTGKLPVPWPTLVTTLTYGVQCFSRYPKHMKRHDFFKSAMPKGYVQERTISFKKDGKYK
TRAEVKFEGRTLVNRIKLKGRDFKEKGNILGHKLRYNFNSHKVYITADKRKNGIKAKFKIRH
NVKDGSVQLADHYQQNTPIGRGPVLLPRNHYLSTRSKLSKDPKEKRDHMVLLEFVTAAGIK
HGRDERYKGA

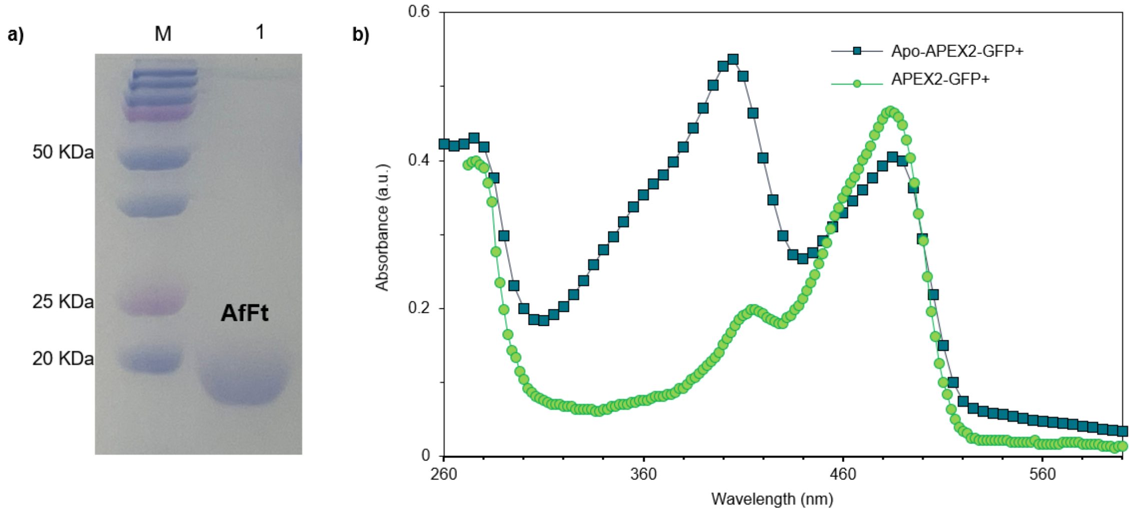

Figure S1. (a) Coomassie-stained 12% SDS-PAGE of AfFt. (b) Absorbance spectrums of 10 μM APEX2-GFP+ enzyme before and after hemin incorporation.

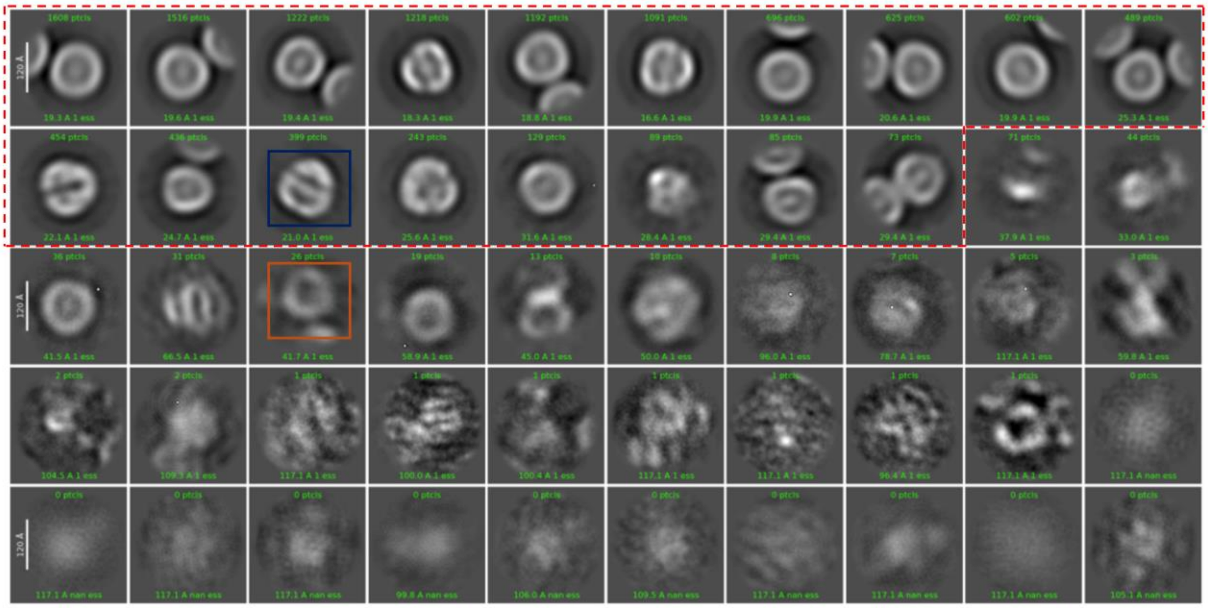

Figure S2. 2D classification results of single-particle reconstruction data for AfFt@. A total of 12,461 particles were initially picked, among which 12,177 particles (enclosed within the red dashed box) were selected for subsequent 3D reconstruction. Each panel shows a representative 2D class average image with its corresponding particle count labelled. The top-left inset in Figure 1e (displayed as the blue-boxed class average) represents AfFt@, containing 399 particles, while the top-right inset (orange-boxed class average) corresponds to empty AfFt, consisting of 26 particles.

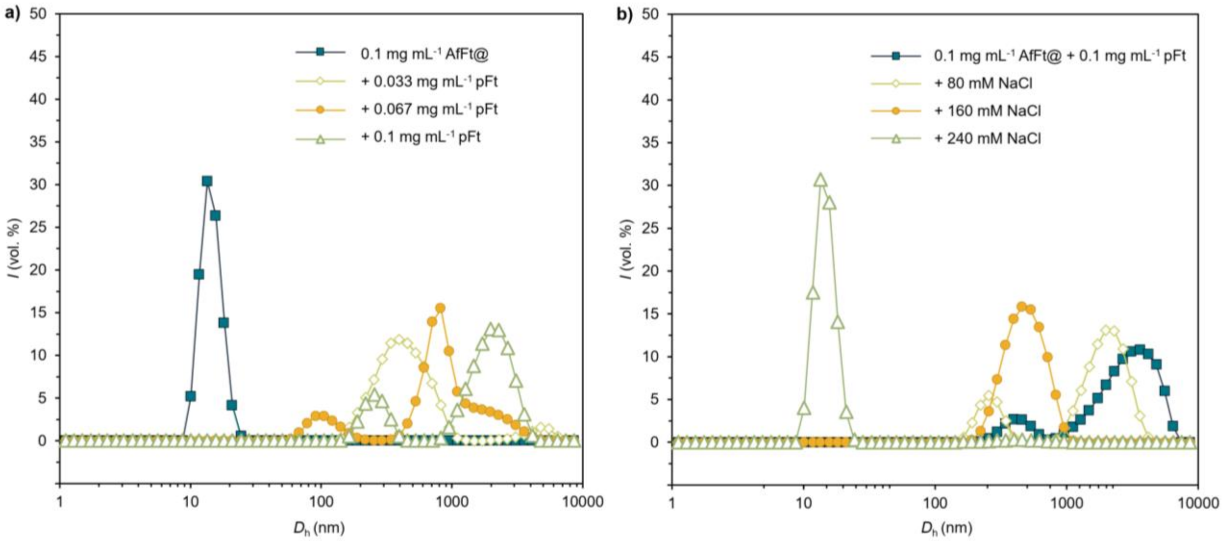

Figure S3. Self-assembly of AfFt@-pFt complexes at pH 7.4. (a) Dynamic light scattering (DLS) data for the volume-averaged size distribution of AfFt@ titrated with an increasing amount of pFt and (b) the resulting complexes disassembled with NaCl.

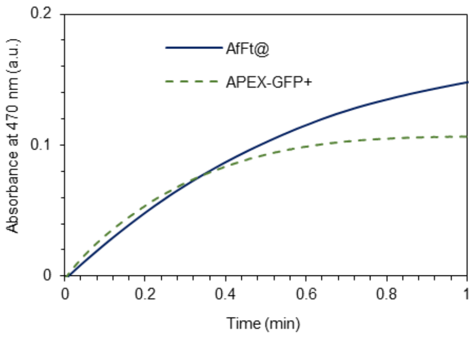

Figure S4. Monitoring the product formation in 10 μM APEX-GFP+ and 10 μM AfFt@, each with 1 mM 2-methoxyphenol and 90 μM H_2O_2, showing the characteristic evolution of absorbance at 470 nm.
